# Re-imagining Research: A Bold Call, but Bold Enough? Comment on "Experience of Health Leadership in Partnering with University-Based Researchers in Canada: A Call to ‘Re-Imagine’ Research"

**DOI:** 10.15171/ijhpm.2019.139

**Published:** 2019-12-22

**Authors:** Bev J. Holmes

**Affiliations:** ^1^Michael Smith Foundation for Health Research, Vancouver, BC, Canada.; ^2^Simon Fraser University, Burnaby, BC, Canada.; ^3^University of British Columbia, Vancouver, BC, Canada.

**Keywords:** Research Partnerships, Co-production, Integrated Knowledge Translation, Health Systems, Complexity

## Abstract

Many articles over the last two decades have enumerated barriers to and facilitators for evidence use in health systems. Bowen et al’s article *"Response to Experience of Health Leadership in Partnering with University-Based Researchers: A Call to ‘Re-imagine Research’"* furthers the debate by focusing on an under-explored research area (health system design and health service organization) with an under-studied stakeholder group (health system leaders), by undertaking a broad program of research on partnerships, and, based on participant responses, by calling for re-imagining of research itself. In response to the claim that the research community is not providing expertise to this pressing issue in the health system, I provide four high level reasons: partnerships mean different things to different people, our language does not reflect the reality we want, our health systems have yet to fully embrace evidence use, and complexity is easier to talk about than act within. Bowen et al’s study, and their broader program of research, is well-placed to explore these issues further, helping identify appropriate researcher-health system leader partnership models for various health system change projects. Given the positive shifts identified in this study, and the knowledge that participants demonstrate about what needs to change, the time is right for bold action, re-imagining not only research, but healthcare, such that the production and use of evidence for better health is embraced and supported.

Confused about the gap between the production of health research evidence and its use in practice? One need only turn to the literature for an (over?) abundance of articles that attempt to explain it. Barriers and facilitators from the perspective of multiple stakeholders have been enumerated in many a journal over the last two decades. One could honestly ask: is there really anything more to say?

Sarah Bowen and colleagues think so, and I am inclined to agree. “*Response to Experience of Health Leadership in Partnering with University-Based Researchers: A Call to ‘Re-imagine Research’*”^1^ reports on semi-structured interviews to explore research-related challenges – but there are new twists to an old approach. First, it focuses on an under-explored research area (health system design and health service organization) with an under-studied stakeholder group (health system leaders). Second, it is one study in a program of research aimed at understanding partnerships. Finally, it does not urge the pursuit of more evidence, calling instead for a re-imagining of research itself.

Indeed, there is something very wrong when “the research community [is not] providing expertise to many important activities that the healthcare system is taking to improve health services” (p. 1). How can this be, especially given the number of talented researchers working in this important field of inquiry?^2^ Below I provide four reasons, based on Bowen et al, mine and others’ work, and my experience as a researcher and leader of a funding agency. Ultimately, I argue that more than research needs re-imagining if citizens are to benefit from better healthcare.

## Partnership Means Different Things to Different People

The authors paint a picture of two worlds at odds: lack of utility of academic research for health systems; a mismatch between academic preparation and health system needs; different motivations of stakeholders; and more. Given this unsatisfactory situation, partnerships between researchers and research users sound like a solution worth pursuing.

However, “partnerships” is too vague a term, as the interviews reveal. Health system leaders describe a range of relationships, from those with researchers who approach them out-of-the-blue with an idea, to closer collaborations borne of mutual interests. The authors themselves note that “partnership” can be variously categorized, from formal traditions such as participatory action research, to developing traditions such as integrated knowledge translation, to simply ways of working, for example collaboration. As Jull et al note,^3^ there are similarities among these areas, but also important differences, and clarity is critical in the selection of an approach.

Why? First, because otherwise assumptions will be made about what the partnership means in practical terms, and expectations will be built (and based on this research, may not be met) based on those assumptions. Second, the very act of seeking clarity can result in the right decision about a partnership model. Not all evidence needs to be co-produced. Sometimes a researcher’s idea provides evidence that is unexpectedly helpful for a health organization; sometimes research commissioned by a health organization is the right way to go.

Given that the framing of a partnership has huge implications for how it plays out in practice^4^ – for example who makes what decisions, who is involved in which conversations – Bowen et al’s work could help by characterizing different partnership models, including the assumptions behind each and the best fit for purpose.

## Our Language Does not Reflect the Reality We Want

Most decision-makers and researchers would no doubt agree that partnership on health system studies is likely to render the most robust and most usable results. As the authors note, “there is emerging evidence that meaningful knowledge user engagement is a major predictor of research utilization” (p. 1).

However, our language does not position us for equality, a critical success factor in partnerships. Note the above quote, which puts the knowledge user in the passive position of “being engaged” – by the researcher. Additional quotes from the beginning of the article also place the researcher in the driver’s seat:

- “…the many traditions of partnered research…share the common characteristics of meaningful engagement of stakeholders or potential research knowledge users…” (p. 1).
- “…suggested actions for improving engagement with knowledge users” (p. 1).

Given the power of language, it is no wonder that “much research on partnerships is based on assumptions of researcher-driven initiatives” (p. 2), or that research co-production risks falling into an existing scientific paradigm, as opposed to being seen as legitimate and credible in its own right.^5^

Discourse studies have long demonstrated that the way things are constructed in language defines to a great extent their reality.^6^ Russell et al suggest that awareness of framing in language can help expose values, preferences and beliefs, and show how we position audiences and construct problems – and that such increased awareness can lead to alternative framings.^7^

As well as characterizing different partnership models, Bowen et al’s future work could examine the language used about and within research co-production and the assumptions behind it, with a goal of creating the authentic partnerships desired by the study participants and many others.

## Our Health Systems Have Yet to Fully Embrace Evidence Use

The purpose of a health research system is to promote, restore and maintain the health of populations.^8^ But it cannot do this alone. Bowen et al’s study suggests to me that a major reason for the research community’s failure to provide expertise to pressing problems is that the health system is not ready for them to do so. “Lack of timeliness, narrowness of focus, and lack of skills in adapting research to a specific context” (p. 4) may be problematic, but they are not the whole story.

Despite research champions within health organizations, Canadian health systems on the whole have an uneasy relationship with research. Some influential people view health research as important but not integral to the delivery of healthcare.^9^ Others see it as a costly and even unethical diversion from patient care.^10^

In England’s National Health Service, research is a core function.^11^ Not so in Canada, as evidenced in this study. Although the authors wanted to focus on research partnerships that address health system design and health service organization, they did not find many. This research for the most part will be instigated by health system leaders, not researchers. What will it take to make it happen?

I argue it will require a health-system-wide commitment to the production and use of research evidence for better healthcare. A “top-down” endorsement and expectation – from the highest levels of the system (ie, governments) – would complement the research-related activity currently underway in healthcare. For example participants talked about embedded researchers and funding programs; some have support from organizational leaders; many are exploring the synergies among quality improvement, evaluation and research for better decision-making. There is much to build on, but it is inconsistent, and seems to be based on organizational leaders’ backgrounds, comfort and preferences. A system-wide commitment in the form of expectations (for example in health organization mandate letters, or via accreditation), and endorsement (for example flexibility of resources, establishment of supportive policies) is necessary.

Bowen et al’s work underscores that the practice and the study of healthcare improvement needs further integration.^12^ Future work would ideally incorporate theories and frameworks from the health research capacity building literature towards not only addressing health system issues, but developing much-needed evidence about research partnerships.

## Complexity Is Easier to Talk About Than Act Within

Study participants demonstrated high awareness of the multi-system change required for effective research partnerships. Indeed, co-production of evidence for health system change will not succeed without addressing many longstanding issues related to how research is conducted, and how its results are used. Most of these issues are complex, in that they involve a range of stakeholders, organizations and sectors with various accountabilities, responsibilities, allegiances, power and influence; despite good intentions, perverse incentives and power dynamics often interfere.^13^

There is much written about complexity and much talk about it among those who work on health and research system change^14^; still, many attempts at system change attempt to simplify complexity rather than work within it.

In an earlier article,^13^ colleagues and I proposed actions for those leading improvement initiatives as well as, importantly, for those with influence on context in which those initiatives are undertaken. Increasingly, I am convinced that more of us need to take leadership in complex systems change: as well as fulfilling responsibilities under our control, we must also work to influence the things we cannot control, but that need to be done.

Bowen et al’s paper describes some of these things – many underway. Academic and healthcare leaders are changing culture within their institutions, challenging assumptions, and collaborating to change mechanisms that impede progress. Funding agencies are offering flexible programs, rethinking conditions for awards and advocating for change in academic and practice settings.^15^ Researchers and clinicians are producing and using evidence in new ways. There are also champions within governments.

By generating evidence related to research partnerships, Bowen et al’s work will ideally shed light on what needs to be re-imagined beyond research, and build momentum, excitement and commitment for the system change that is needed.

## Summary

Researcher-health system partnerships have potential, but as Bowen et al note, the specific benefits – and how those benefits are best achieved – are little understood. Given the positive shifts identified in this study, and the knowledge that participants demonstrate about what needs to change, the time is right for bold action. Further exploring assumptions and models of partnerships and language, urging change and setting expectations at the highest levels, and committing to working with complexity, will help us re-imagine not only research, but healthcare, such that the production and use of evidence for better health is embraced and supported.

## Ethical issues

Not applicable.

## Competing interests

Author declares that she has no competing interests.

## Author’s contribution

BJH is the single author of the paper.
